# Basis statistics for phylogenetic tree shape: tree balance formulas beyond expectations

**Published:** 2026-10-29

**Authors:** Daniel J. Bauman, Noah A. Rosenberg

## Abstract

Summary statistics that measure the shapes of phylogenetic trees contributed to quantitatively understanding evolutionary processes. Among phylogenetic summary statistics, the tree balance indices have been designed to capture intuitive notions of balance or imbalance in trees. Here, we characterize the tree balance indices in terms of basis statistics, mapping the exponentially large space of rooted, binary, unlabeled trees to lower-dimensional spaces. We observe that many tree balance indices can be represented in terms of linear functions of these low-dimensional spaces, enabling us to compute exact formulas for the means, variances, and covariances of many balance indices under the Yule--Harding model. The new framework recapitulates existing formulas for the expectations of 8 indices: Sackin index, average leaf depth, total cophenetic index, $\hat{s}$-shape statistic, Colless index, corrected Colless index, quadratic Colless index, and rooted quartet index for binary trees. It produces new formulas for the expectations of 9 additional indices: equal weights Colless index, total $I$ index, total $I'$ index, Rogers $J$ index, total internal path length, total path length, average vertex depth, stairs1 index, and stairs2 index. Moreover, the framework also allows us to calculate the exact variance of all 17 indices that are represented by the bases as well as the covariance and correlation for all $\binom{17}{2} = 136$ pairs among the 17 indices.

